# A theoretical rethinking of ecosystem services from the perspective of social-ecological system

**DOI:** 10.1016/j.isci.2025.112309

**Published:** 2025-03-28

**Authors:** Wei Jiang, Rainer Marggraf

**Affiliations:** 1 State Key Laboratory of Regional and Urban Ecology, Research Center for Eco-Environmental Sciences, Chinese Academy of Sciences, No.18 Shuangqing Road, Haidian District, Beijing 100085, China; 2 Department of Agricultural Economics and Rural Development, University of Goettingen, Heinrich-Düker-Weg 10, 37073 Goettingen, Germany

**Keywords:** Environmental science, Nature conservation, Ecology

## Abstract

Ecosystem service (ES) research has grown rapidly, but emergent concepts such as disservices, supply-demand, relationships, and flows remain fragmented. An increasing consensus emphasizes that ecosystems cannot deliver services without human inputs, positioning ES as coproducts of coupled social-ecological systems (SES). This necessitates a theoretical rethinking of ES concepts from an SES perspective to advance comprehensive ES science. Through the SES lens, ES quantity and value are redefined as interactions between ecosystem supply and human demand. ES relationships are clarified by distinguishing inherent bundle characteristics from SES-level equilibria, which underpin cross-system flows. The framework integrates these advances by grounding ES in human-ecosystem interdependence, linking supply-demand dynamics to ES realization, resolving ambiguities in relationships through equilibrium analysis, and framing flows as outcomes of SES equilibria. By unifying these concepts, the framework addresses current inconsistencies and charts future research priorities: optimizing supply-demand balance, analyzing SES equilibria mechanisms, and modeling cross-system flow pathways.

## Graphical abstract

**Figure:**
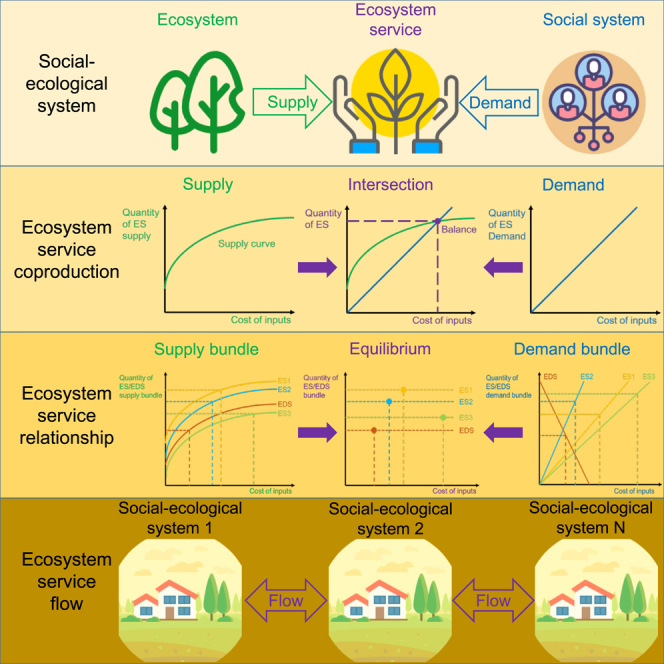

## Introduction

The concept of ecosystem service (ES) originates from the collaboration between ecology and economics,^1^ which can be traced back to the late 1970s and the early 1980s, when the terms “nature’s services”^2^ and “ecosystem services”^3^ were proposed in 1977 and 1983, respectively. Without explicit definitions, both terms were more such as a metaphor for ecosystem functions. In 1997, two seminal publications coined the ES concept: Daily placed more emphasis on ecosystems by defining ES as “the conditions and processes through which natural ecosystems, and the species that make them up, sustain and fulfill human life”^4^; whereas Costanza et al. proposed the widely acknowledged definition of ES, “the benefits human populations derive, directly or indirectly, from ecosystem functions”, implying the distinction between ecosystem functions, ecosystem services, and benefits.^5^

The large program Millennium Ecosystem Assessment (MA) in 2005 was a major step forward to a global recognition, in which ES was straightforwardly defined as the benefits people obtain from ecosystems, including supporting, provisioning, regulating, and cultural services.^6^ MA also developed the first conceptual framework that saw ES as a bridge between nature and human well-being.^6^ After MA, the understanding of this concept had since been intensively discussed, such as the distinction between intermediate and final services,^7^ means and ends of services,^8^ and active and passive utilization of ecosystems.^9^ This discussion inspired the proposal of the cascade framework, which considers ES as a bridge that provides the link from ecosystem structures, processes, and functions in the ecological realm, through ES, and to benefits and values in the social realm.^10^^,^^11^ This cascade framework was later integrated into the second conceptual framework developed by The Economics of Ecosystems and Biodiversity.^12^

Before TEEB, two important concepts had been introduced. The concept of ecosystem disservices (EDS) was initially introduced in the research on agriculture in 2007,^13^^,^^14^ highlighting the negative effects of nature on human well-being.^15^ The concept of ES relationships had drawn attention since 2009, including ES trade-off and ES synergy that describe the antagonistic or win-win situations between ES, as well as ES bundle that describes the repeatedly appearing sets of ES across space or time.^16^

Rooted in the MA, the concepts of ES supply and demand have been recognized in the first half of the 2010s. On the one hand, a number of similar terms have been proposed to describe ES supply based on biophysical properties, ecological functions, and social properties, such as ES capacity,^17^ ES provision,^18^ managed supply of ES,^19^ as well as potential supply and capability of ES.^20^ On the other hand, the demand for ES has been elaborated into three perspectives: the actual use or consumption of an ES,^17^ the amount of an ES required or desired by society,^21^ and the expression of an individual’s preferences for specific attributes of an ES.^22^ The difference between the quantities of supply and demand was deemed the mismatch of ES,^19^ while the spatial connection between supply and demand was described as the flow of ES.^23^ Further, ES supply, demand, and flow were expanded to be service providing areas, service benefiting areas, and service connecting areas.^24^

The establishment of the Intergovernmental Science-Policy Platform on Biodiversity and Ecosystem Services (IPBES) in 2012 marked the mainstreaming of the concept into global governance and policy-making. The IPBES developed the third conceptual framework, in which the concept of nature’s contributions to people was put forward to represent all the contributions, both positive and negative, of living nature to the people’s quality of life, aiming to embrace diverse worldviews and knowledge systems on human–nature relations and to cover beneficial and detrimental contributions of nature to people.^25^^,^^26^

The exponential growth of ES studies over the past decades has resulted in the emergence of three conceptual frameworks as well as various ES-related concepts, including ES valuation,^27^^,^^28^ ES mapping,^29^ EDS,^30^ ES supply,^31^ demand^32^ and flow,^33^ and ES relationships.^34^ These concepts have found wide applications in food security, biodiversity conservation, land use planning, and environmental governance and policy-making.^35^ However, ES-related concepts have not yet been completely integrated into existing conceptual frameworks, thus lacking a consistent theory that logically connects the concepts of EDS, ES supply-demand, ES relationships, and ES flows. This leads to the consequences that those concepts are used in a reciprocally independent manner and remain a source of confusion and misunderstanding. Specifically, the understanding of the correlation between ES supply-demand and ES relationship remains ambiguous; for example, studies on ES relationship and ES supply-demand are separate and independent,^36^ while it is argued that trade-offs exist not only in ES but also in ES supply and demand.^37^

An increasing consensus has been reached that ecosystems per se cannot deliver any service to people without human inputs, including human capital, social capital, and built capital.^27^^,^^38^^,^^39^^,^^40^ Hence, ES can be deemed coproducts of a coupled social-ecological system (SES).^41^^,^^42^^,^^43^ A theoretical rethinking of ES concepts from the perspective of SES is thus timely necessary and supportive for taking the first step toward a higher level of comprehensive ES science. Therefore, the purposes of this study are to clarify the nature of ES, to integrally conceptualize the coproduction of ES based on the supply from the ecological realm and the demand from the social realm, and to develop a theoretical framework that connects the important ES-related concepts including ES supply, demand, bundle, relationship, and flow in a logic and consistent way.

## The nature of ecosystem services

The nature of ES has long been perplexed by the association among ecosystem functions, ES, and human benefits, experiencing the development from the partial unification of ecosystem functions and ES,^2^^,^^3^^,^^4^ through implicit distinction among ecosystem functions, ES, and benefits,^5^^,^^6^^,^^10^^,^^12^ to another partial unification of ES and benefits.^26^^,^^44^ Here, we suggest a triune interpretation of ES through an analogy between ecosystems and man-made machines and a thought experiment of the world before Homo sapiens arise.

First, we consider a washing machine. It generally composes of a variety of components and realizes the main function of cleaning clothes through various physical processes such as warming, rolling, and shaking, and so forth. Through this function, people obtain the service of washed clean clothes, thereby gaining the benefit of saving time and labor for manually washing clothes. This implies that the function, service, and benefit actually refer to the same thing. The essential distinction between the function and service provided by a washing machine lies in the necessary human inputs, such as putting clothes into the washing machine, adding enough detergent, and taking clothes out of it, which thus reflect people’s demand for the function of washing machines.

We then imagine the natural world before Homo sapiens arose. In a world without human beings, different natural ecosystems, such as rainforests, prairies, and savannas and so forth. performed their inherent functions such as timber growth, water retention, and gas regulation and so forth based on various physical, chemical, and biological processes. After Homo sapiens arose, humans not only learned to utilize these functions through their own inputs, such as fruit harvest, timber logging, or wildlife hunting and so forth but also told stories about a forest, recreated at a river bank, and drew pictures of animals. They gradually realized that some ecosystem functions such as gas regulation, soil and water conservation, and protection from extreme events and so forth were also helpful and necessary for their living and surviving. Ecosystem functions and services are distinguished by whether they are needed by humans. The need for ecosystem functions is reflected by human investments such as labor, time, and all kinds of other resources. The benefits humans obtain from ecosystems are exactly those multiple ES.

Based on both of the analogy and the thought experiment, we can conclude that ecosystem functions are the overall performance of an ecosystem, including an array of geochemical, physical, and biological processes that occur within the ecosystem,^45^ while ES can be defined as the ecosystem functions that are demanded by people and the beneficial contributions from ecosystems to people. This definition has four advantages. First, it unifies ecosystem functions, ES, and human benefits by explicitly introducing human demand. For example, pollination is an ecosystem function that is fundamental to the reproduction and persistence of flowering plants.^46^ This function has been pursued and thus recognized as an ES for centuries since farmers imported and managed colonies of European honeybees to fields and orchards.^47^ The pollination service is the benefit people obtain, without which the persistence of ecosystems would be challenging, if not impossible.^46^ Second, it provides a consistent interpretation of ES and EDS. By definition, EDS can be understood as the ecosystem functions that are disliked by people and the detrimental contributions from ecosystems to people, which is analogous to an economic bad. Third, it is open to potential ecosystem functions that have currently not been recognized but will probably be recognized as ES if they are demanded by people in the future. Fourth, it avoids the concepts that describe the “potential” or “ability” of ecosystems for providing services, which are unclear to define, difficult to measure, and indistinguishable from the actual supply of ES.

## Ecosystem service coproduction by ecosystem supply and human demand

**Figure 1:**
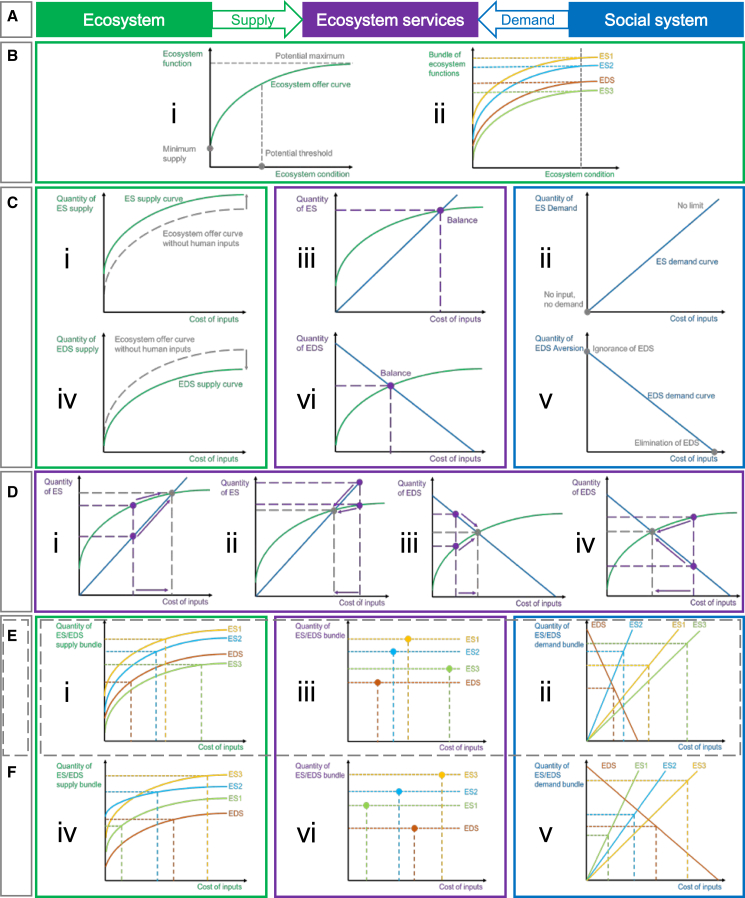
Coproduction of ecosystem services from both of ecosystem and social system (A) Ecosystem services link the ecological subsystem and the social subsystem through supply and demand, building a coupled social-ecological system. (B) Ecosystem offer curves are constructed for a single ecosystem function and a bundle of ecosystem functions. (C) The interactions between supply and demand curves determine the quantity of an ecosystem service/disservice at a certain cost of inputs. (D) The dynamics underlie the process from imbalance to balance of the supply of and demand for an ecosystem service/disservice. (E) The trade-offs through the cost of inputs on the supply and demand bundles of an ecosystem result in the partial equilibrium of a social-ecological system. (F) The trade-offs through the cost of inputs on the supply and demand bundles of multiple ecosystems result in the general equilibrium of a social-ecological system. Green color represents the supply side of the ecosystem, the blue color represents the demand side of the social system, and the purple color represents the coproduction of ecosystem service.

In analogy to microeconomics, ES and EDS can be understood as the interactive outcome between the supply from ecosystems and the demand from humans (Figure 1A). Studies have reported that biodiversity substantially affects and determines ecosystem functions,^48^^,^^49^^,^^50^ and the focus on biodiversity has been expanded to ecosystem characteristics,^51^ ecosystem integrity,^52^ and ecosystem conditions.^53^ Ecosystem conditions refer to the quality of an ecosystem measured in terms of its abiotic and biotic characteristics, which underpin the integrity of the ecosystem and support the performance of ecosystem functions.^53^ Hence, a given ecosystem function can be delineated by a function of ecosystem conditions.

We describe the function between the ecosystem function and the ecosystem conditions by depicting an ecosystem offer curve derived from the following characteristics: (1) an ecosystem in extremely low conditions, such as a degraded grassland,^54^ provides a minimum of ecosystem functions, determining the starting point of the ecosystem offer curve on the y axis; (2) an ecosystem function has an upper limit that is constrained by the gross primary production,^55^ indicating the progressive trend of the ecosystem offer curve to the potential maximum; (3) the ecosystem offer curve is monotone increasing because the improvement of ecosystem conditions leads to the increase in ecosystem function; (4) the impacts of ecosystem conditions on the ecosystem function is non-linear^50^ based on the assumption that a small improve of ecosystem conditions results in an accelerated increase in ecosystem function, which then levels off after a threshold of ecosystem conditions, indicating the inverse-L shape of the ecosystem offer curve (Figure 1Bi).

It has generally been recognized that direct and indirect drivers are able to alter ecosystem conditions, thus changing the supply of ES.^12^^,^^25^^,^^53^ People often influence ecosystem conditions in a targeted way by protecting or restoring ecosystems and so forth rather than taking them for granted so that more units of ecosystem functions are provided. Ecosystem conditions thus depend on human inputs, including all forms of resources such as time, energy, materials, and labor, and so forth, which can generally be measured in terms of monetary costs. Ecosystem functions, by definition, are the supply of ES and EDS, meaning that the supply of ES and EDS can further be represented as a function of the costs of inputs. Hence, the supply curves of ES and EDS are constructed by vertically moving the ecosystem offer curves: the supply curve of ES moves upward with the cost of inputs (Figure 1Ci), while the supply curve of EDS moves downward (Figure 1Civ).

As mentioned above, human inputs reflect people’s demand for ES and their aversion for EDS, implying that the demand for ES and the aversion for EDS can also be expressed by human inputs measured in terms of costs. We also describe the function between the demand/aversion and input costs by depicting a demand curve of ES and an aversion curve of EDS. The demand curve of ES is constructed based on the characteristics: (1) no human input indicates no demand for a certain service, suggesting the starting point of the demand curve on the origin; (2) more units of ES are considered better for people, implying that the demand for ES generally has no limit; (3) The demand curve is monotone increasing because the demand increases along with growing inputs; (4) for simplicity, the demand curve is supposed to be linear (Figure 1Cii). In comparison, the aversion curve of EDS is illustrated based on the characteristics: (1) an EDS is at the maximum when no human input has been invested, implying people’s ignorance of the EDS; (2) the aversion curve is monotone decreasing because people are willing to invest resources to reduce EDS; (3) sufficient invested resources can eliminate EDS, indicating that the aversion curve ends on x axis; (4) for simplicity, the aversion curve is also supposed to be linear (Figure 1Cv).

The intersection between supply and demand/aversion curves and their dynamic processes determines the quantity of a certain ES provided by a certain ecosystem and the amount of the cost of inputs at the balance (Figure 1Ciii). For example, if the quantity of supply is larger than the quantity of demand at a given cost of inputs, decision makers for ecosystem management and beneficial stakeholders have motivations to raise the investments so that the quantity of both supply and demand increases to the balance (Figure 1Di). In contrast, if the quantity of supply is smaller than the quantity of demand at a given cost of inputs, decision makers for ecosystem management and beneficial stakeholders have motivations to reduce the investments so that the quantity of both supply and demand decreases to the balance (Figure 1Dii). The interpretation of EDS is similar (Figures 1Cvi, 1Diii, and 1Div).

## Ecosystem service relationships within a social-ecological system

An ecosystem provides a variety of functions, generating a bundle of ecosystem functions, e.g., including three types of ES and one EDS. At a given level of ecosystem condition, the quantity of individual ecosystem function (ES1, ES2, ES3, and EDS) is different (Figure 1Bii). Instead of the terms of trade-off and synergy, we adopt the terms of substitute and complement derived from microeconomics as inherent characteristics of an ecosystem function bundle, which describe antagonistic and win-win relationships between ecosystem functions. The substitute relationship refers to the case that an increase of an ecosystem function leads to the decrease of another ecosystem function, for example, the increase of crop production usually leads to the decrease of carbon sequestration^36^; whereas the complement relationship refers to the case that an increase of an ecosystem function leads to the increase of another ecosystem function, for example, the improvement of pollination leads to better crop production.^46^

We adopt the term of trade-off to explicitly answer the questions of who makes trade-offs and through which medium trade-offs are made. Confronted with budget constraints, decision makers and beneficial stakeholders have to make trade-offs by adjusting invested resources on both of supply and demand sides within a SES. The trade-offs made among multiple ES/EDS of a single ecosystem within a SES (Figure 1Ei, Eii) generates the result of partial equilibrium of the SES (Figure 1Eiii), while the trade-offs made among various ES/EDS bundles provided by different ecosystems within a SES generates the result of general equilibrium of the SES (Figure 1Eiii and 1Fvi). For example, a certain SES involves multiple types of ecosystems such as a forest, a cropland, and a lake. If all ES provided by the forest are traded off to the balance between corresponding supplies and demands, whereas the ES provided by other ecosystems remain unbalanced, this SES reaches partial equilibrium. If all ES provided by every individual ecosystem are traded off to the balance, this SES reaches general equilibrium. Both partial and general equilibrium are the fundamental features of an SES.

## Ecosystem service flows among multiple social-ecological systems

**Figure 2:**
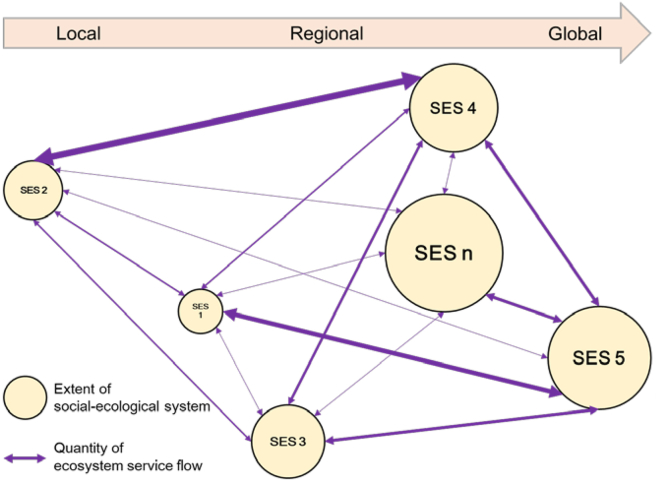
Ecosystem service flows connect multiple social-ecological systems at local, regional, and global scales

ES flows are generally understood as spatial connections between supply and demand.^33^ From the perspective of an SES, such an ES flow is actually the quantity of ES determined by the interaction between supply and demand, thus being an internal stuff within the SES. Based on the concepts of partial and general equilibriums developed above, we suggest a new interpretation of ES flows. After the ES within a SES have been traded off to reach partial or general equilibriums of the SES, there are still ES flowing out of the SES into another SES owing to spillover effects.^56^ For example, in the global trade system, food-exporting countries (e.g., Brazil) export agricultural products (e.g., soybean) to food-importing countries (e.g., China).^57^ For this reason, these ES flows can be interpreted as externalities and connections among multiple SES at local, regional, and global scales. Such externalities can be either positive or negative, depending on the overall quantity of ES and EDS (Figure 2).

Box **Key concepts and definitions**

**Social-ecological system**: Integrated systems that link the supply from the ecological subsystem and the demand from the social subsystem to generate ecosystem services.

**Ecosystem condition**: the quality of an ecosystem measured in terms of its abiotic and biotic characteristics, underpinning the integrity of the ecosystem and supporting the performance of ecosystem functions.

**Ecosystem function**: The overall performance of an ecosystem, including an array of geochemical, physical, and biological processes that occur within the ecosystem. The quantity of an ecosystem function depends on ecosystem conditions, which is described by an ecosystem offer curve.

**Ecosystem service**: The ecosystem functions that are demanded by people and the beneficial contributions from ecosystems to people, including supporting, provisioning, regulating, and cultural services. **Ecosystem disservice:** The ecosystem functions that are refused by people and the detrimental contributions from ecosystems to people.

 **Supply of ecosystem service**: The quantity of supply that is affected by all forms of human inputs measured in terms of costs, which is described by a supply curve.

 **Demand for ecosystem service**: The quantity of demand that is reflected by all forms of human inputs measured in terms of costs, which is described by a demand curve.

 **Balance between supply and demand**: The intersection of supply and demand curves determines the quantity of an ecosystem service and the costs of human inputs. A dynamic process will force an imbalance to reach a new balance.

**Ecosystem function bundle**: A variety of functions generated by different combinations of geochemical, physical, and biological processes. A given level of ecosystem conditions determines differentiated quantities of individual ecosystem functions, which is described by the ecosystem offer curves. Three inherent characteristics are identified in an ecosystem function bundle.

 **Substitute**: An increase of an ecosystem function leads to the decrease of another ecosystem function.

 **Complement**: An increase of an ecosystem function leads to the increase of another ecosystem function.

 **Neutral**: A change in an ecosystem function does not affect the change in another ecosystem function.

**Ecosystem service trade-off**: The quantities of individual ecosystem services are traded off by adjusting human inputs on both the supply and demand sides in the context of budget constraints. Trade-offs result in partial and general equilibrium, which are fundamental characteristics of a social-ecological system.

 **Partial equilibrium**: The result of trade-offs among multiple ecosystem services of a certain ecosystem.

 **General equilibrium**: The result of trade-offs among various ecosystem services provided by different ecosystems.

**Ecosystem service flow**: The externalities of ecosystem services between social-ecological systems at different spatial scales.

## Implications of the understanding of ecosystem services

**Figure 3:**
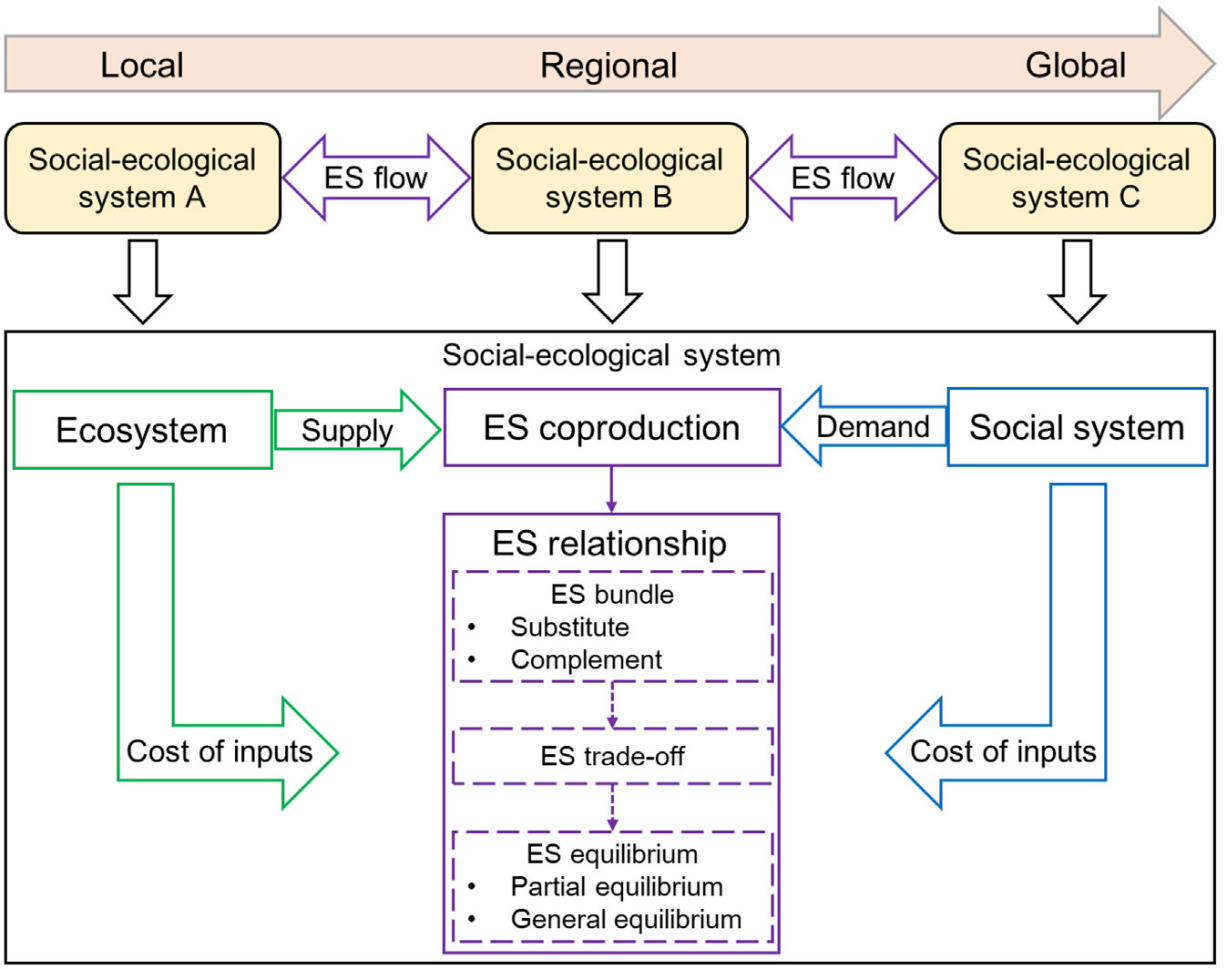
The conceptual framework developed in this study

We summarize the illustrations above to construct a conceptual framework from the perspective of SES (Figure 3). This framework provides a consistent logic to cover the important progress on ES-related concepts, including EDS, ES supply-demand, ES relationships, and ES flows. By suggesting a triune definition of ES that unifies ecosystem function, ecosystem service, and human benefit, ES and EDS can be interpreted by a common logic, providing the first level of consistency. By explicitly introducing the element of human demand, the quantity and value (in monetary terms) of ES/EDS are jointly determined by ecosystem supply and human demand, their dynamic process until the balance implies the second level of consistency. ES/EDS relationships are distinguished between the inherent characteristics of ES bundles and partial/general equilibria of the whole social-ecological system. The third level of consistency lies in the connection between the balance of supply-demand of one ES/EDS and partial/general equilibria of multiple ES/EDS in the whole SES through trade-off. The partial/general equilibria of a social-ecological system further build the basis of ES flows as connections among multiple social-ecological systems, indicating the fourth level of consistency between ES relationships and ES flows.

The reconceptualization of ES from the perspective of SES has several implications. First, the thought experiment indicates the chronological origins of provisioning services, cultural services, and regulating services, respectively, implying that the origins of multiple ES provided by certain ecosystems can be identified by archaeological discovery, historical documents, and traces of human activities, and so forth. For example, the people living along the Weser River in Germany have created various manifestations, thanks to the cultural ES of the river.^58^ Second, the interaction between ES/EDS supply and demand/aversion provides an insight for the valuation of ES/EDS. For example, the costs of inputs at the balance can be interpreted as the lower bound of the value of an ES or the higher bound of the negative “value” of an EDS. Third, the new understanding of trade-offs can be used to identify the boundary of an individual SES. For example, a group of people and a certain ecosystem constitute a coupled system if those people are able to affect the ES/EDS supply or demand by investing resources. Fourth, trade-offs among various ES/EDS bundles of different ecosystems are helpful for explaining global land use changes. For example, the trade-off from regulating services of forests to provisioning services of croplands is partly responsible for the deforestation in the Amazon rainforests,^59^ while the trade-off from provisioning services of croplands to regulating services of forests partly explains the afforestation in the Chinese Loess Plateau.^60^ Fifth, the interpretation of ES flows as the externalities of a SES implies that a potential payment for the ES scheme should be based on the difference between the inflow and outflow of the SES. Finally, providing new and more reliable measures of ES is a highly worthwhile challenge because of their great potential for offering important insights into the functioning of coupled SES and for supporting environmental policy-making and regional planning.

## Conclusions

This study introduces a rethinking of ES as coproducts of a SES, delving into how ES/EDS are determined by the intersection between ecosystem supply and human demand. The conceptual framework proposed in this study offers a clear logic that connects the emergent ES-related concepts, including ES supply-demand, ES relationships, and ES flows, thus filling a significant gap in current studies. This theoretical and systemic thinking would make contributions to the development of interdisciplinary fields between ecology and social sciences.

The framework provides theoretical support and practical guidance for future research. Three directions can be considered from the supply side of ES, the demand side of ES, and the whole SES. First, given the diversity and complexity of ecosystem functions, the future research on the supply side needs to identify the underlying processes and measurable indicators of individual ecosystem functions and thus to empirically reveal the functions between ecosystem functions and conditions. Temporal aspects bring additional complexity to this quantification. The effects of human interventions on ecosystem conditions on the quantity of ecosystem functions should be further addressed before ES supply can be quantified with confidence. Second, great challenges remain in the quantification of the demand side. This study provides a new insight for estimating demand for ES by taking the costs of human inputs into account, which requires innovative methodology to empirically determine the dependence of ES demand on the cost of inputs. Third, another enormous challenge is to develop a feasible approach for quantitatively addressing partial and general equilibria among multiple ES within a SES and then to test the hypothesis that the equilibria status is positively correlated to resilience^61^ and sustainability^62^ of the SES. When equilibriums in multiple SESs have been quantified, the spatial flows of ES between these SESs can be calculated, providing a practical basis for designing payment for ES schemes.

## Acknowledgments

This study was supported by the 10.13039/501100001809National Natural Science Foundation of China (72394401).

## Declaration of interests

The authors declare that they have no known competing financial interests or personal relationships that could have appeared to influence the work reported in this article.
